# Accuracy of robotic-assisted implant placement in second molars combined with step drills and manual-automatic switching function: a retrospective study

**DOI:** 10.1186/s12903-025-07227-0

**Published:** 2025-11-26

**Authors:** Tao Yang, Xiaojian Xing, Wenan Xu, Buling Wu

**Affiliations:** 1 Shenzhen Clinical College of Stomatology, School of Stomatology, Southern Medical University, Shenzhen, China; 2 Shenzhen Stomatology Hospital (Pingshan) of Southern Medical University, No. 143, Dongzong Road, Pingshan District, Shenzhen, 518118 Guangdong China

**Keywords:** Accuracy, Second molar, Robotic-assisted, Implant placement surgery, Step drills

## Abstract

**Background:**

Nowadays, robotic computer-assisted implant surgery (r-CAIS) has drawn great attention in implant dentistry; however, the clinical application of r-CAIS for limited operating space such as the second molar region, has not been demonstrated. Thus, this study aimed to evaluate the accuracy of r-CAIS in second molars in combination with step drills and manual-automatic mode switching function.

**Methods:**

Patients who lost second molars and underwent r-CAIS were enrolled in this study. The positioning marker was installed preoperatively, and a cone-beam computed tomography (CBCT) scan was subsequently performed. The CBCT data were subsequently transferred into the robotic software, and a preoperative surgical plan was generated. After marker registration and calibration, the implant osteotomy was completed by the robotic arm under the control of the surgeons. Owing to the limited operating space in the second molar region, the operation mode of r-CAIS was switched between manual and automatic modes, and step drills were used in combination. Patients underwent postoperative CBCT, and the accuracy was evaluated using the CBCT data between the planned and placed implants.

**Results:**

A total of 16 patients were enrolled in this study, and no adverse events occurred during or after surgery. The mean global coronal, apical and angular deviations were 0.43 ± 0.10 mm (95%CI: 0.38 to 0.49 mm), 0.44 ± 0.10 mm (95%CI: 0.38 to 0.49 mm), and 0.88 ± 0.53°(0.60 to 1.16°), respectively.

**Conclusions:**

r-CAIS can achieve high accuracy in the second molar region when combined with step drills and manual-automatic mode switching function. Further prospective studies are needed to validate the effects.

## Background

Owing to the very posterior location of the second molars, maintaining proper oral hygiene in this area is challenging. Additionally, second molars are often affected by impacted wisdom teeth, leading to severe caries [1], root resorption [2, 3], and periodontitis [4], thus contributing significantly to the prevalence of tooth loss in this region.

The management of missing second molars remains controversial in clinical practice. Previous study demonstrated restoration of a missing second molar with an implant-supported crowns could increase both masticatory ability and patient satisfaction [5]. Following second molar loss, approximately 20% of unopposed teeth develop 2 mm super-eruption, but there was no clear correlation between excessive eruption and occlusal interference [6]. Restoration should be considered when patients report compromised mastication or desire to maintain dental arch integrity [6]. Available treatment modalities include removable partial dentures, fixed partial dentures, and implant-supported prostheses. However, while the cross-arch design required for removable dentures compromises patient comfort, fixed prostheses inevitably introduce unfavourable mechanical stresses due to their cantilevered single-end configuration. Therefore, dental implants emerge as an appropriate option, offering good biomechanical advantages and patient satisfaction [7].

The accuracy of implant placement is closely linked to the long-term stability of both soft and hard tissues [8, 9], and the emergence of computer-assisted technologies has enabled surgeons to position implants with greater precision compared to freehand implantation [10, 11]. Currently, computer-assisted technologies include static computer-assisted implant surgery (s-CAIS), dynamic computer-assisted implant surgery (d-CAIS), and robotic computer-assisted implant surgery (r-CAIS).

A clinical study utilized s-CAIS in implant surgery for patients undergoing second molar implants, achieving a high level of accuracy [12]. Nevertheless, s-CAIS presents several limitations, including heat injury because of the guide plate, the lack of flexibility in changing the plan intraoperatively, and deviations arising from the gaps between the guide plates and drills [13–15]. d-CAIS has been applied in various clinical situations and has achieved great accuracy [16, 17], however, reports on the application of d-CAIS in second molars remain scarce.

The advent of r-CAIS has further advanced the development of oral implantology. The precision of r-CAIS has been substantiated by various in vitro and in vivo studies [18, 19]. According to a retrospective clinical study, the accuracy of r-CAIS was significantly greater than that of s-CAIS, with coronal, apical and angular deviations of 0.43 ± 0.18 mm versus 1.31 ± 0.62 mm; 0.56 ± 0.18 mm versus 1.47 ± 0.65 mm, and 1.48 ± 0.59° versus 2.42 ± 1.55°, respectively [19]. A model study demonstrated that r-CAIS was more precise than d-CAIS at both fresh extraction sites and healed single tooth sites [20].

**Fig. 1:**
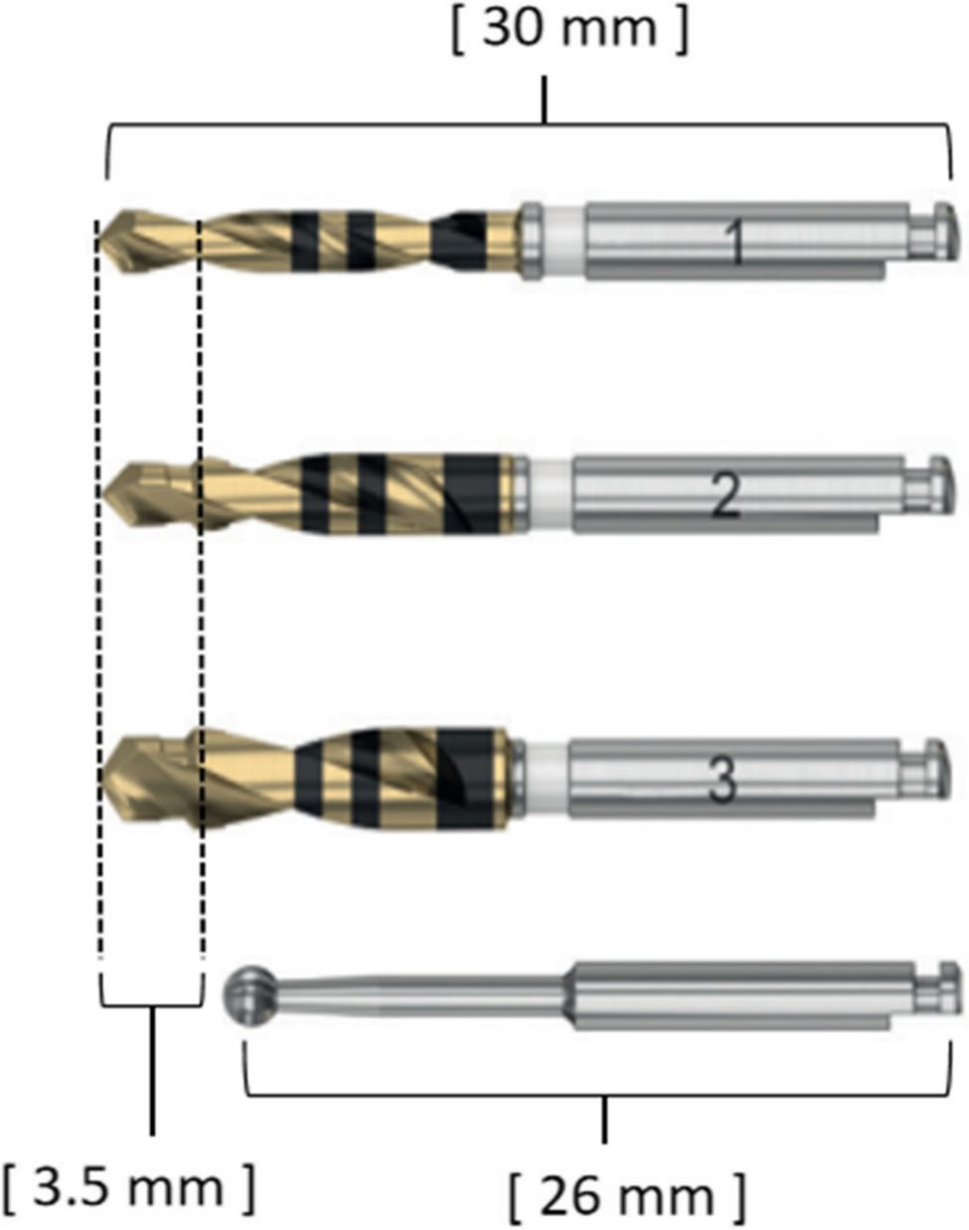
Illustration of the step drill and round bur

The application of s-CAIS and d-CAIS in the second molar region is currently constrained by limited operating space. Although r-CAIS holds promise for use in this domain, researchers have also noted challenges related to the difficulty in applying this technology in the second molar region [21]. To date, few studies have reported on the utilization of r-CAIS in the second molar region. In a case series comprising 10 cases of robotic-assisted single-tooth implantation, only two involved posterior teeth, specifically the first molars [21]. Based on our previous clinical practice, we observed that r-CAIS can be switched between automatic and manual modes. In automatic mode, the procedure is performed independently by the robotic arm. This mode is viable in areas with adequate space but encounters difficulties in confined surgical sites where movement may be impeded by the opposing dentition. In manual mode, all joints of the robotic arm are unlocked, allowing the surgeon to freely manipulate the implant handpiece for optimal surgical access. Combined with step drills (specialized drills featuring a tip diameter smaller than the body diameter, as shown in Fig. 1), it is possible to apply r-CAIS in the second molar region, even with limited operating space. Therefore, this study aims to investigate the accuracy of r-CAIS in the second molar region in combination with step drills and manual-automatic mode switching function.

## Methods

### Study design and population

**Fig. 2:**
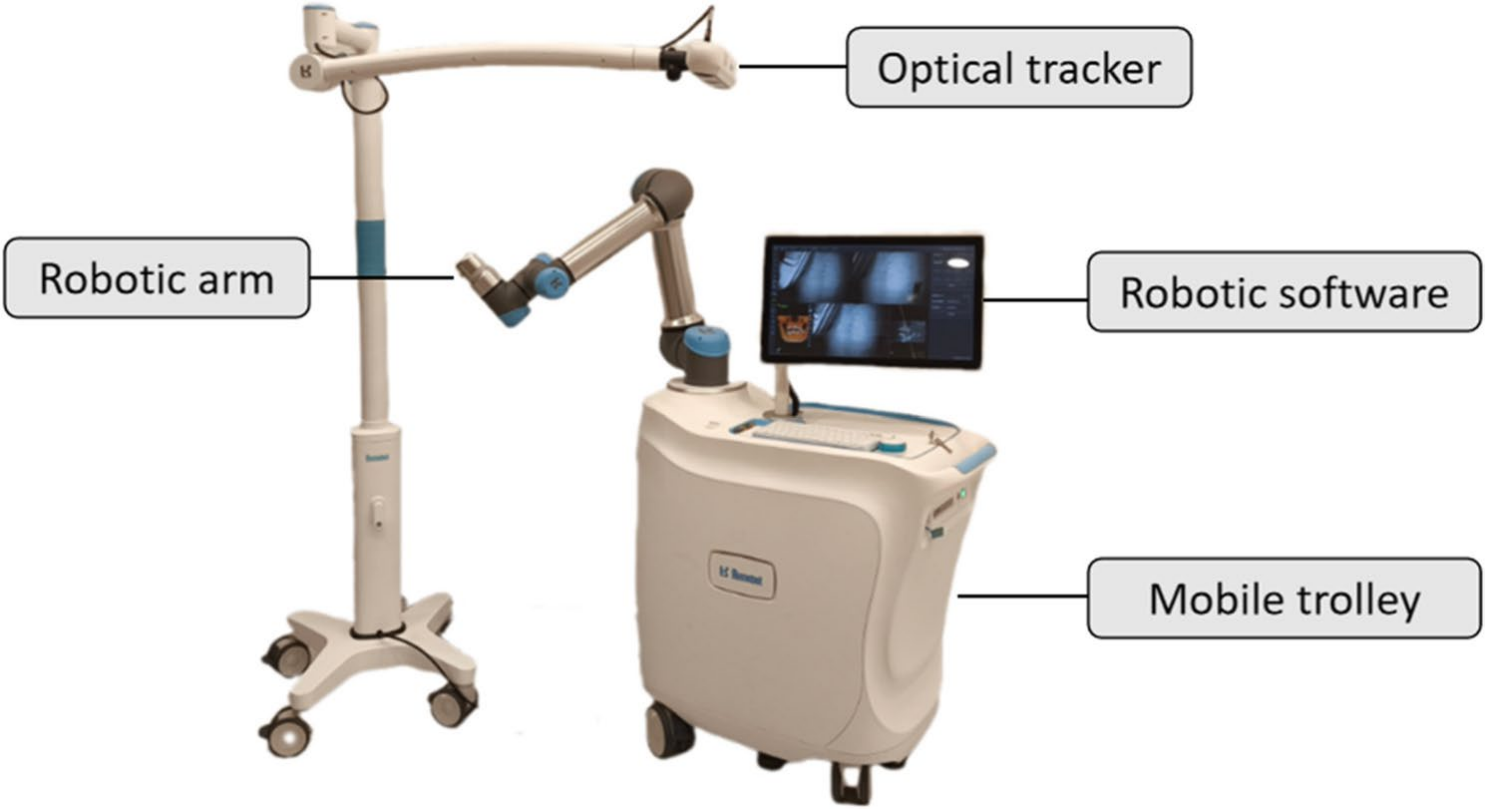
The structure of the r-CAIS system

This study presented a retrospective case series of r-CAIS for the second molar region, and the protocol was approved by the local Ethics Committee of Shenzhen Stomatology Hospital (Pingshan) of Southern Medical University (No. 202412 A). The patients were chosen from the specialized records of the Department of Implant Dentistry. Only patients who underwent implant placement surgery in the second molar region using the Remebot robotic system (Baihui Weikang, Beijing, China; Fig. 2) between June 2023 and May 2024 were included in the study.

The inclusion criteria were as follows:

(1) single second molar absence or consecutive posterior teeth absence within a second molar; (2) patients with limited operating space who are unable to complete the process of the robotic automatic mode; (3) patients undergoing r-CAIS combined with step drills; (4) patients with good general and periodontal health; (5) light smoking (< 10 cigarettes/day) or no smoking; and (6) good treatment compliance.

The exclusion criteria were as follows: (1) uncontrolled systemic or periodontal diseases; (2) general contraindications for implant placement surgery; (3) heavy smoking (> 10 cigarettes/day) or alcoholism; (4) acute phase of any disease; (5) severe bruxism or clenching; and (6) psychologically unfit to undergo robotic surgery.

### Workflow of r-CAIS

**Fig. 3:**
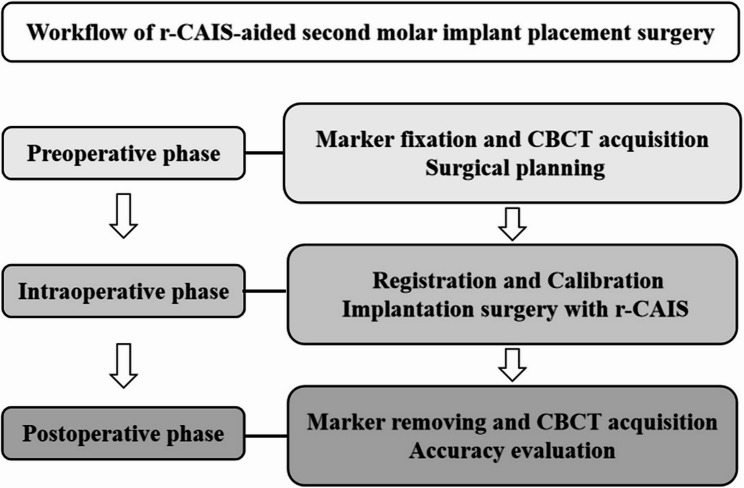
Workflow of r-CAIS-aided second molar implant placement

The workflow of r-CAIS was divided into the preoperative, intraoperative, and postoperative phases, as illustrated in Fig. 3.

#### Preoperative phase

**Fig. 4:**
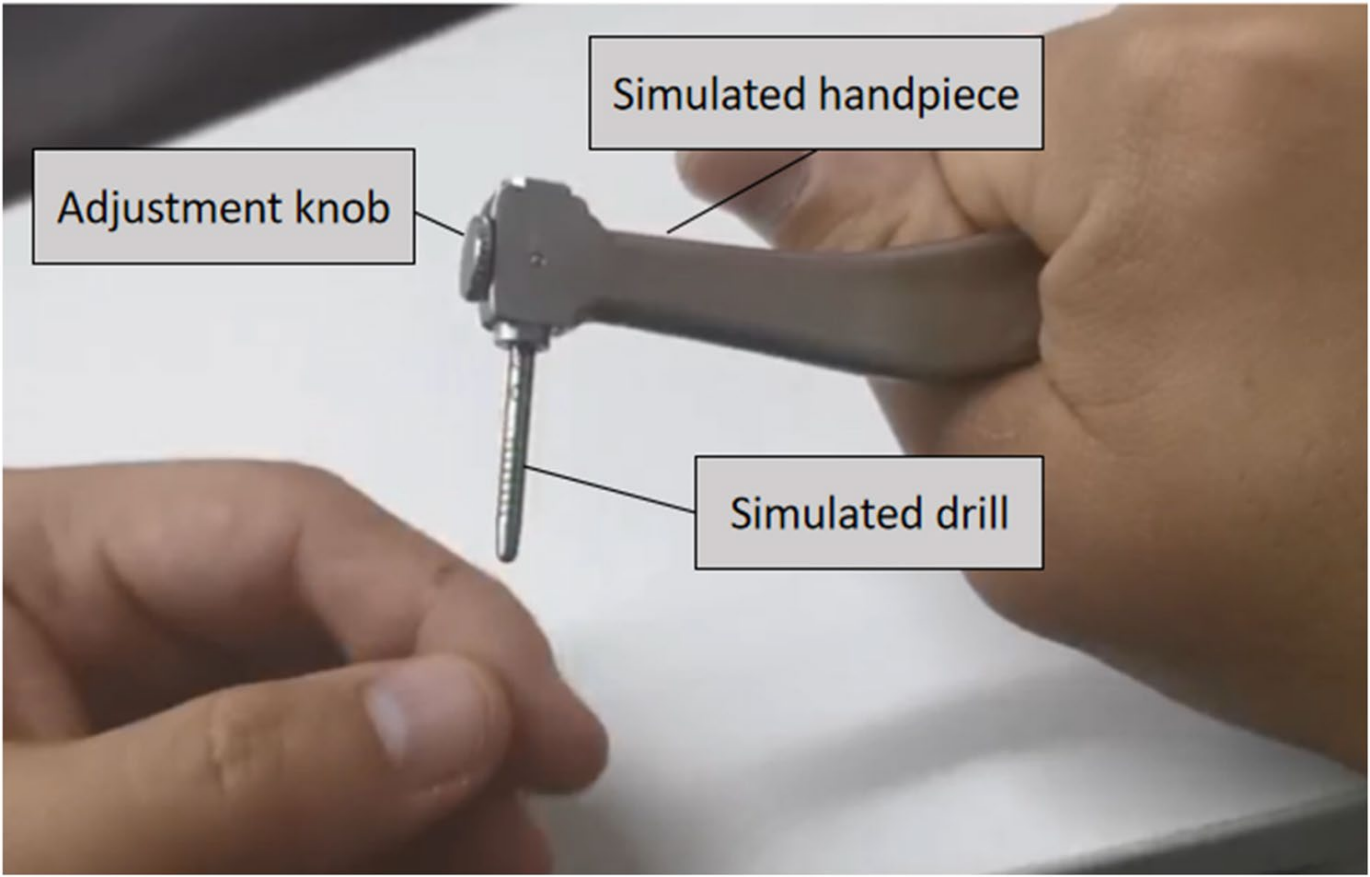
Illustration of the simulated surgical handpiece

**Fig. 5:**
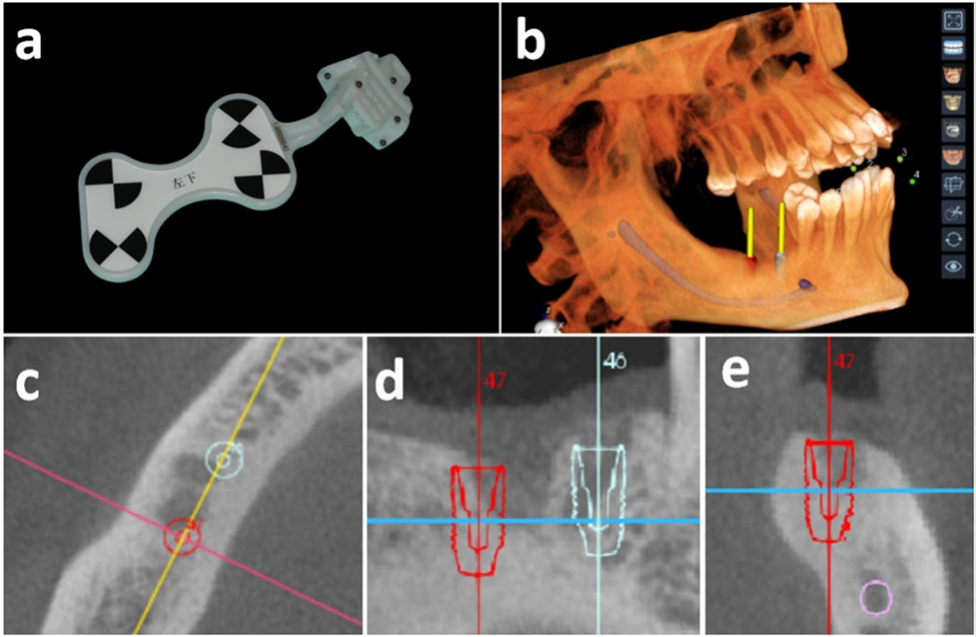
Preoperative surgical plan. (**a**) Positioning marker. (**b**) 3D reconstruction image of the surgical region. (**c**-**e**) Transverse, coronal and sagittal planes of the preoperative design. The right mandibular second molar implant design is marked with a red outline

The implant system we used includes step drills in two lengths (30 mm and 36 mm). The shorter ones are preferred when operating space is limited. Preoperatively, a custom-made simulated implant handpiece was employed to assess the operational space (Fig. 4). This instrument was designed to mimic a surgical handpiece, incorporates a length-adjustable simulated drill. If the simulated handpiece with a 30-mm drill length could not be comfortably positioned in the second molar region during maximal mouth opening, intraoperative mode switching might be required.

r-CAIS system was preheated and calibrated before operation to ensure good functionality. The patients were instructed to gargle with 0.12% chlorhexidine three times, for one minute each time. Based on the surgical site, the opposite teeth (generally cusp teeth and premolars) were chosen as the region to install a universal positioning marker (shown in Fig. 5a). Subsequently, self-curing acrylic resin (Protemp™, 3 M ESPE, Neuss, Germany) was injected into the inner slot of the marker, which was then gently positioned onto the opposite teeth until it solidified. Afterwards, a CBCT (KAVO, Biberach, Germany) scan was performed with parameters set at 120 kV/1.38 mA, a 0.25 mm voxel size, and a scanning time of 26.9 s. The Digital Imaging and Communications in Medicine (DICOM) format data from the CBCT were subsequently imported into r-CAIS software for simulation of the surgical plan, which was confirmed by the surgeons and engineers, as shown in Fig. 5b-e.

#### Intraoperative phase

**Fig. 6:**
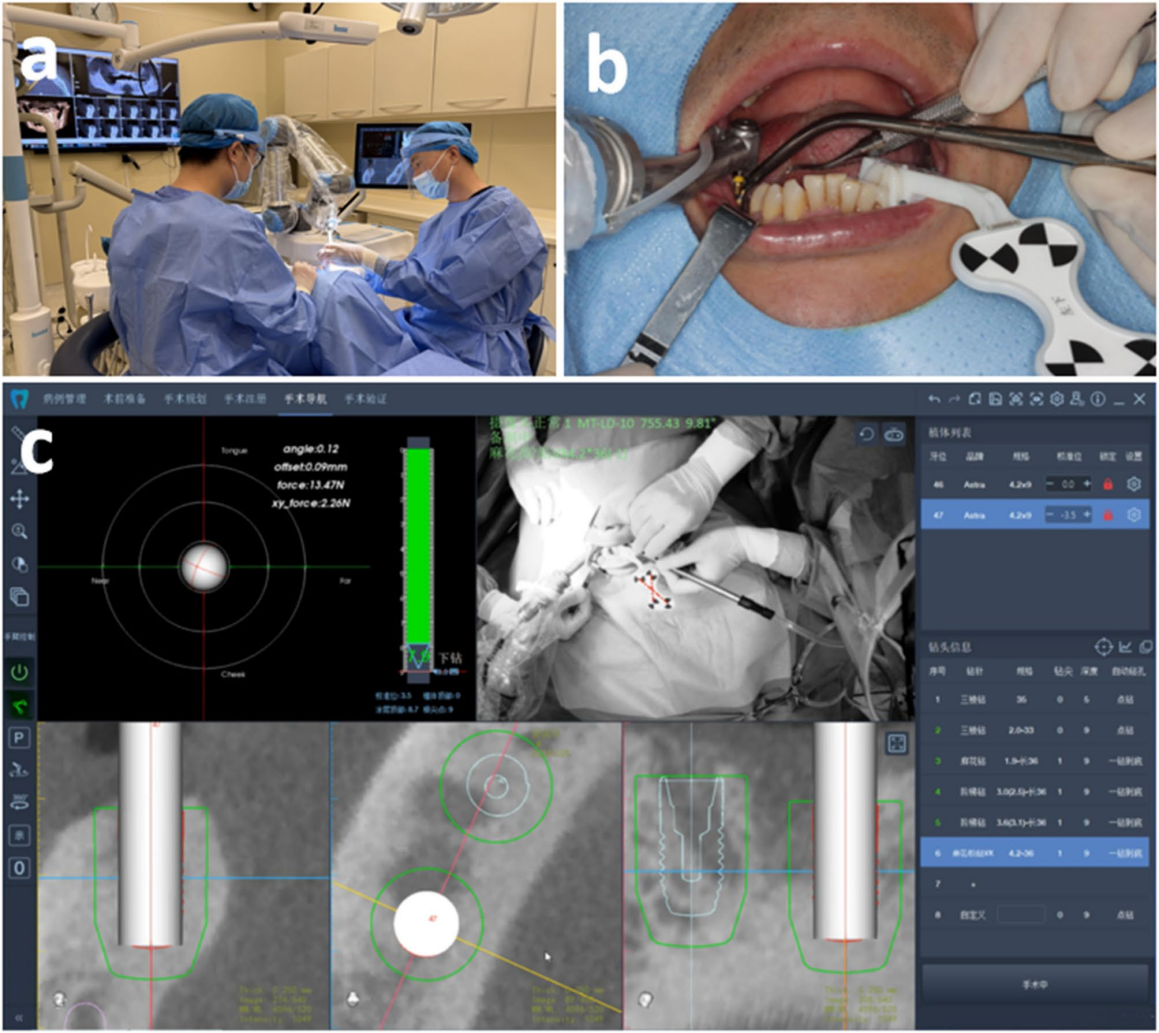
Robotic computer-assisted implant surgery for patient 16. (**a**) Robotic-assisted implant surgery. (**b**) Implant surgery was performed via the robotic arm. (**c**) Real-time feedback image on the computer screen

**Fig. 7:**
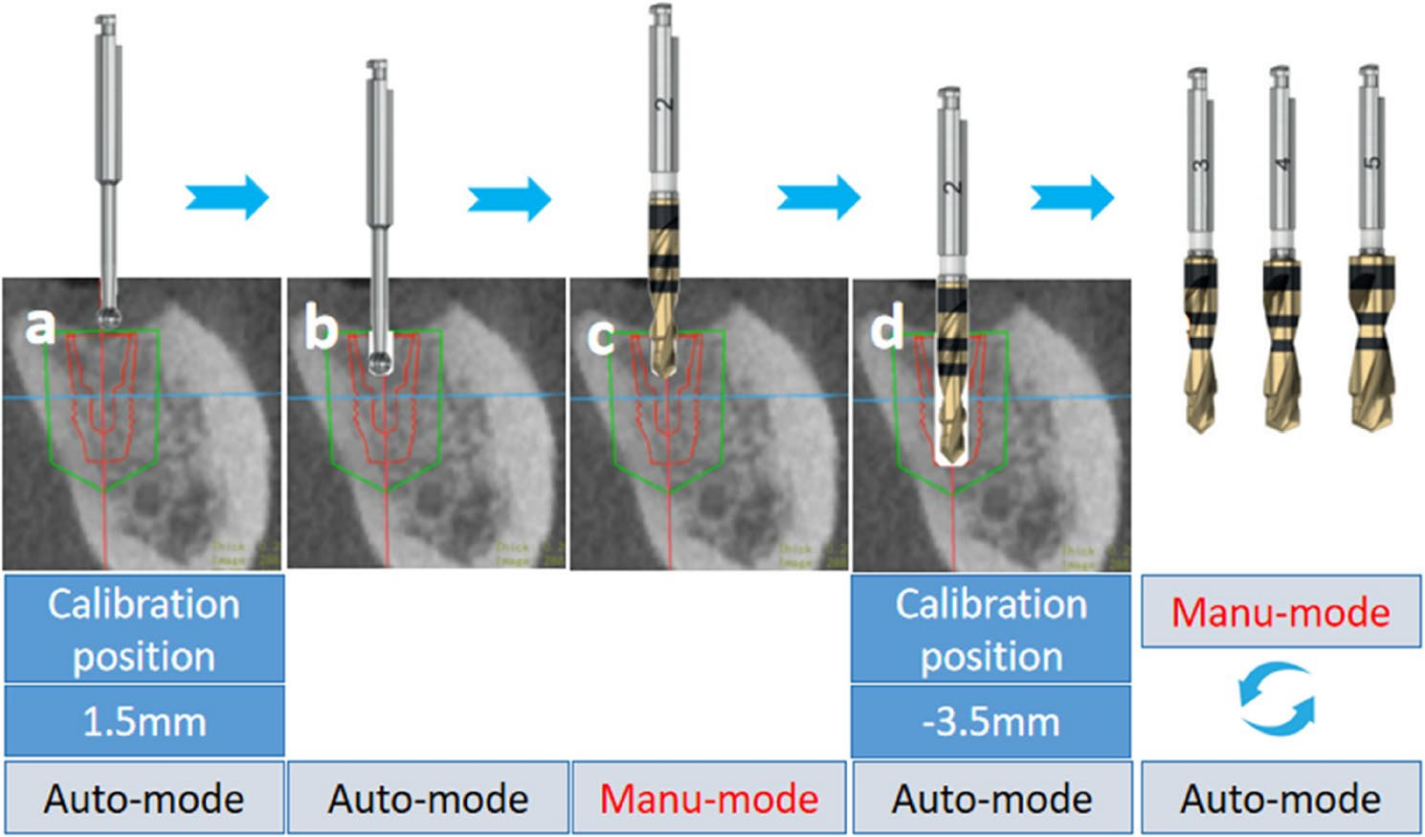
Diagram illustrating the drill sequence and the position of the initial drill point. (**a**) A round bur with the initial position set at 1.5 mm. (**b**) The round bur prepared the osteotomy site to a depth of 3.5 mm in automated mode. (**c**) The apical portion of the step drill was manually inserted into the prepared osteotomy site. (**d**) The step drill completed full-length implant osteotomy preparation in automated mode

The surgical region was disinfected with iodophor both externally and internally, followed by covering with a sterile perforated towel. Local anaesthesia was administered via Primacaine^®^ (4% Articaine, 1:100,000 adrenaline, ACTEON, Mérignac, France). The positioning marker and robotic arm were subsequently registered and calibrated. The operator then moved the robotic arm near the surgical area to start the surgery and supervise the surgery using a real-time feedback computer screen, as shown in Fig. 6. A round bur and a series of step drills (AstraTech OsseoSpeed EV, Dentsply Sirona, Pennsylvania, USA) were used. A short step drill (30 mm in length) was installed at the handpiece to assess whether the surgical handpiece can be smoothly maneuverer to the surgical region in semi-automatic mode without interference from the opposing dentition. If interference persisted, the following protocol was initiated:

- Step 1: In automatic mode (Fig. 7a), under the system’s default initial drill tip-to-osteotomy distance (1.5 mm), a round bur (length: 26 mm; diameter: 1.9 mm) was used to prepare the osteotomy site to a depth of 3.5 mm (Fig. 7b).
- Step 2: The handpiece was switched to manual mode, and the tip of the first short step drill (length: 30 mm; tip diameter: 1.9 mm; body diameter: 2.6 mm) was inserted into the pre-prepared osteotomy site (Fig. 7c).
- Step 3: The initial drill tip-to-osteotomy distance was adjusted to −3.5 mm, after which automatic mode was activated to complete the preparation along the full implant length (Fig. 7d). Subsequently, the manufacturer-recommended drilling sequence was followed, progressively enlarging the osteotomy with step drills of increasing diameters to finalize the implant osteotomy.

The insertion torque value was measured by a dynamometric wrench. When the value was above 15 N.cm, an appropriate healing cap was installed. If it was below 15 N.cm, a covering screw was applied instead. The wound was sutured with 5–0 polypropylene nonabsorbable sutures (Ethicon, Johnson & Johnson, New Jersey, United States).

#### Postoperative phase

Postoperative instructions were provided to the patients, which included refraining from brushing or gargling for 24 h and eating soft foods for one week. They were also instructed to rinse their mouth with 0.12% chlorhexidine twice daily for five days. Additionally, patients were prescribed oral systemic antibiotics (150 mg roxithromycin, administered twice daily for 3 days) and analgesics (200 mg ibuprofen tablets, taken three times daily as needed). The sutures were removed two weeks postoperatively.

#### Evaluation of accuracy

**Fig. 8:**
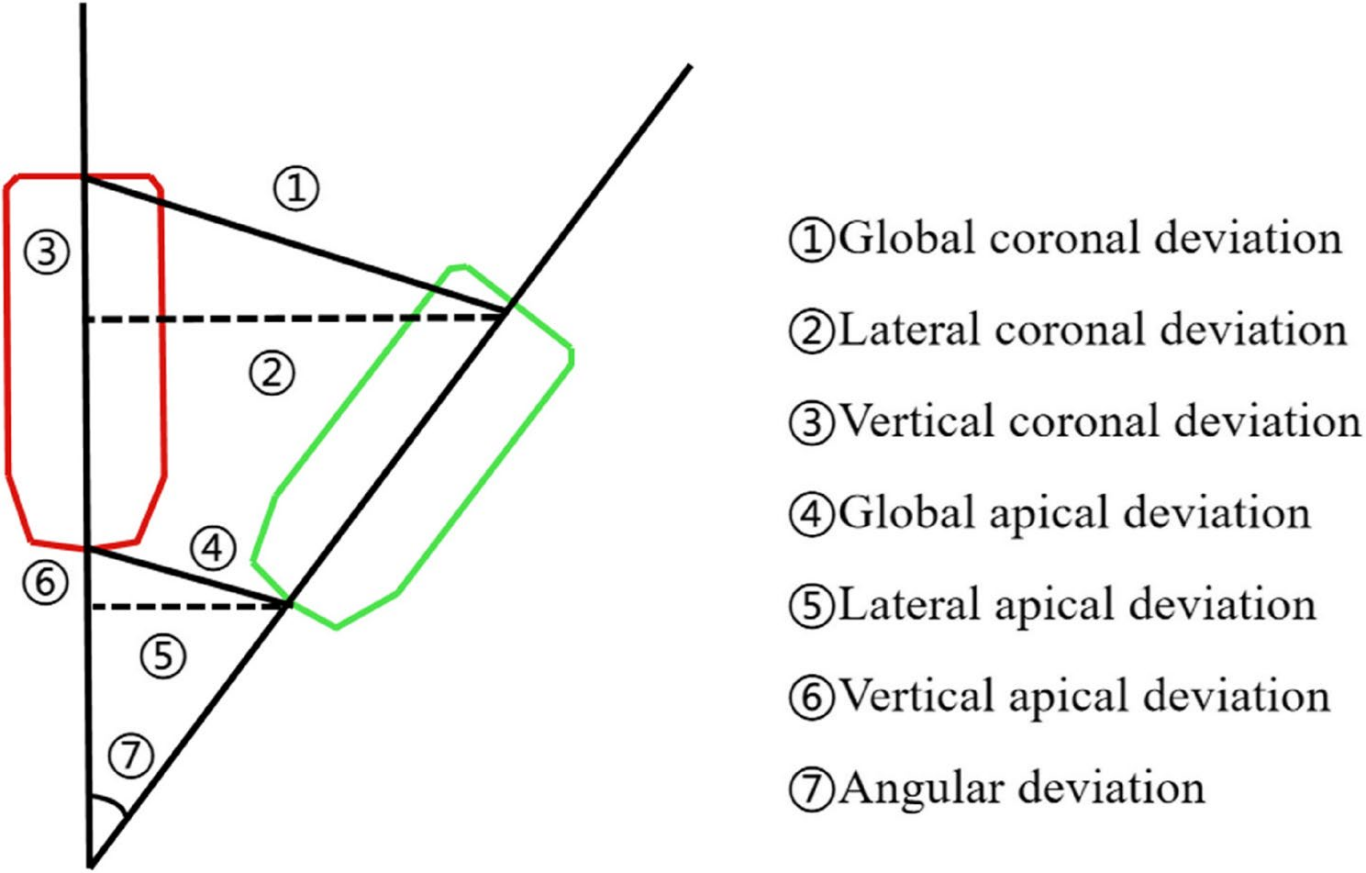
Graphical representation of the deviations between the planned and placed implants

Postoperative CBCT was performed and imported into the r-CAIS software to merge the preoperative and postoperative DICOM data. The deviations were assessed on the central axes of the planned and placed implants, as shown in Fig. 8. An accuracy analysis report was then generated, including coronal (global, lateral, and vertical), apical (global, lateral, and vertical), and angular deviations.

#### Statistical analysis

The quantitative data are expressed as standard descriptive statistics, including means, standard deviations, maximum and minimum values, and upper and lower 95% confidence intervals (CIs). The normality of the variables was assessed using the Shapiro–Wilk test (α = 0.05). Statistical analyses were performed via SPSS 23.0 software (IBM Corp., New York, United States).

## Results

### Patient characteristics

A total of 16 patients, with an age range of 29–60 (mean: 44.3 ± 9.8 years), were enrolled in the study. No adverse events were reported during or after the operation. Table 1 provides an overview of the patients’ demographic and surgical characteristics. Eight patients each lacked a single second molar or consecutive posterior teeth which included a second molar. Four maxillary and 12 mandibular second molars were included. One conical and 15 cylindrical implants were used, with diameters ranging from 4.2 to 4.8 mm and lengths ranging from 8 to 11 mm. Additionally, the insertion torque varied from 5 to 45 N.cm.

**Table 1:** Patient demographic and surgical characteristics

Patient number	Age	Sex	Site	Implant type	Implant size	Implant geometry	Insertion Troque (*N*.cm)	Healing type
1	48	M	#17	Astra EV	4.8s×9	cylindrical	35	transgingival
2	42	M	#17	Astra EV	4.8s×8	cylindrical	25	transgingival
3	44	M	#27	Astra EV	4.8s×8	cylindrical	45	transgingival
4	53	F	#27	Astra EV	4.2s×9	cylindrical	30	transgingival
5	32	F	#37	Astra EV	4.8c×9	conical	45	transgingival
6	53	F	#37	Astra EV	4.2s×9	cylindrical	45	transgingival
7	58	M	#37	Astra EV	4.2s×9	cylindrical	35	transgingival
8	38	F	#47	Astra EV	4.8s×11	cylindrical	25	transgingival
9	54	F	#47	Astra EV	4.8s×9	cylindrical	30	transgingival
10	42	F	#47	Astra EV	4.2s×8	cylindrical	10	non-transgingival
11	29	F	#47	Astra EV	4.8s×9	cylindrical	35	transgingival
12	44	M	#47	Astra EV	4.8s×9	cylindrical	10	non-transgingival
13	30	F	#47	Astra EV	4.8s×11	cylindrical	10	non-transgingival
14	34	F	#47	Astra EV	4.8s×9	cylindrical	35	transgingival
15	60	M	#47	Astra EV	4.2s×11	cylindrical	5	non-transgingival
16	49	F	#47	Astra EV	4.2s×9	cylindrical	10	non-transgingival

*M* male, *F* female

### Accuracy

**Fig. 9:**
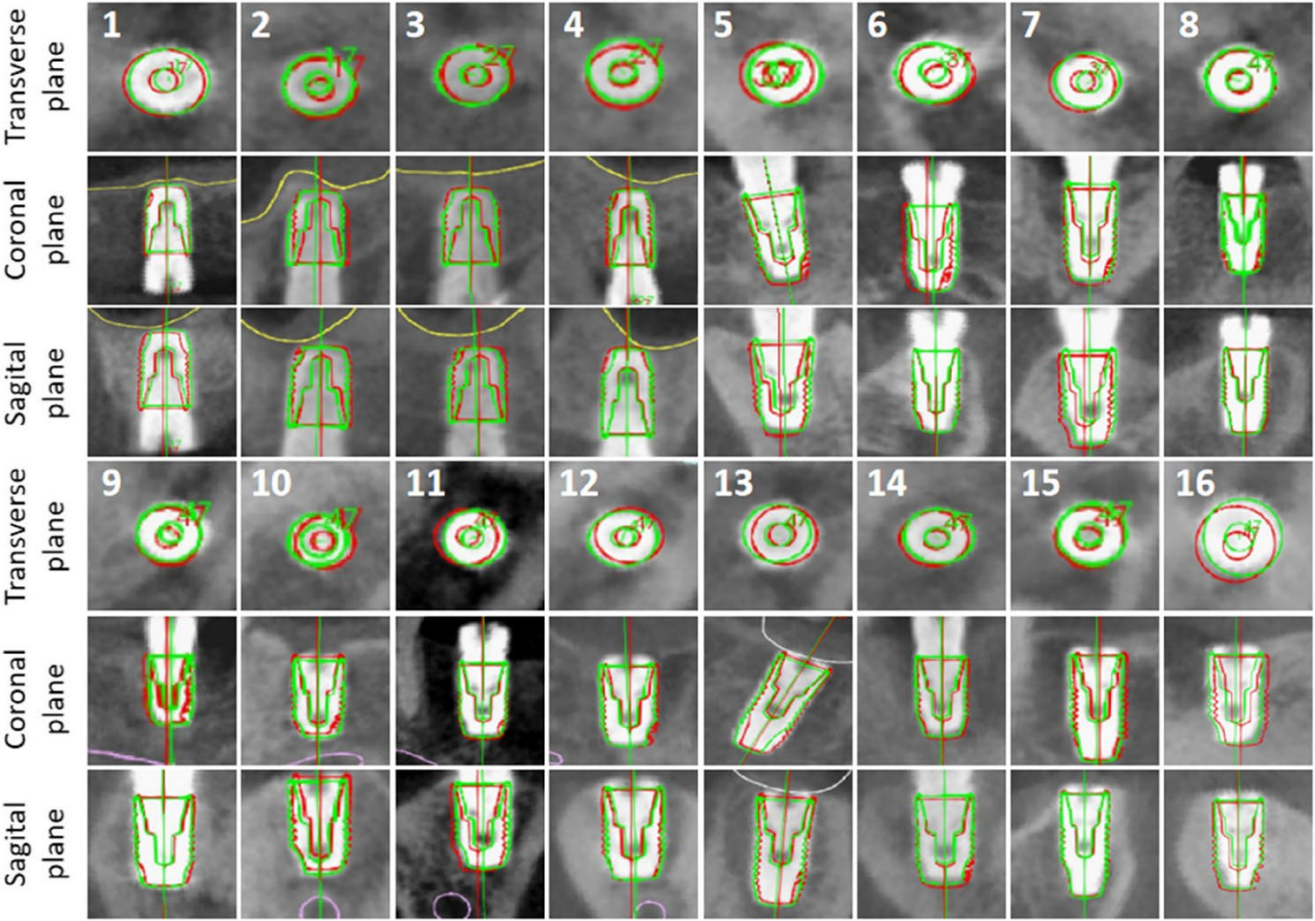
CBCT fusion images of the planned and placed implants for each patient. The planned implants are outlined in red, and the placed ones are outlined in green

**Fig. 10:**
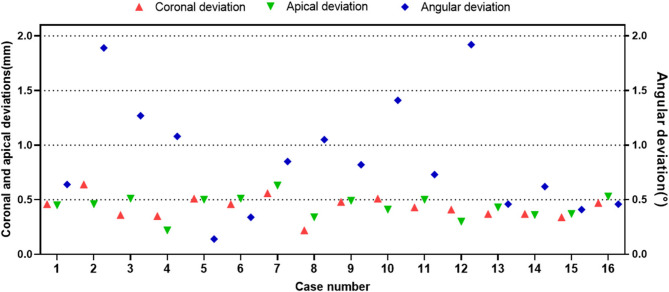
Global coronal, apical, and angular deviations for each case

Figure 9 shows the CBCT fusion images for each patient. The quantitative outcomes are listed in Table 2. The mean global coronal, apical and angular deviations were 0.43 ± 0.10 mm (95%CI: 0.38 to 0.49 mm), 0.44 ± 0.10 mm (95%CI: 0.38 to 0.49 mm), and 0.88 ± 0.53°(0.60 to 1.16°), respectively. All data conformed to a normal distribution via the Shapiro–Wilk test (*p* > 0.05). Figure 10 shows the coronal, apical, and angular deviations of each case.

**Table 2:** Descriptive statistics of implant deviations

Deviation	Mean	SD	Median	Min	Max	L95% CIs	U95% CIs	Shapiro-Wilk test
Global coronal deviation	0.43	0.10	0.45	0.22	0.64	0.38	0.49	0.94
Lateral coronal deviation	0.35	0.11	0.34	0.17	0.62	0.30	0.41	0.73
Vertical coronal deviation	−0.04	0.25	−0.09	−0.45	0.39	−0.18	0.09	0.59
Global apical deviation	0.44	0.10	0.46	0.22	0.63	0.38	0.49	0.80
Lateral apical deviation	0.36	0.12	0.35	0.14	0.51	0.29	0.42	0.20
Vertical apical deviation	−0.04	0.25	−0.09	−0.45	0.39	−0.18	0.09	0.58
Angular deviation (◦)	0.88	0.53	0.78	0.14	1.92	0.60	1.16	0.22

*CIs* Confidence intervals, *SD* Standard deviation

## Discussion

To the best of our knowledge, the application of the robotic system’s manual-automatic switching function combined with step drills in the second molar region was reported for the first time. The results demonstrated high accuracy, with mean global and apical deviations of less than 0.5 mm and a mean angular deviation of less than 1°. The results of this study suggest that r-CAIS may serve as a promising navigation tool for implant placement in the second molar region.

In recent years, r-CAIS has demonstrated high accuracy in various clinical applications, including single-tooth, immediate, edentulous, and zygomatic implant placement [21–24]. These findings are consistent with ours and may be attributed to their inherent performance characteristics. First, the robotic arm exhibits a high level of repeatability accuracy. According to the manufacturer’s data, the repeatability error is measured at 0.033 mm [21]. Additionally, when subjected to varying resistance changes, the robotic response time can reach 0.06 s [8]. The characteristics above collectively ensuring exceptional precision.

Owing to the limited operating space, the implementation of s-CAIS and d-CAIS in the second molar region presents significant difficulties. The application of s-CAIS presents difficulties due to its size, along with the longer drills used [25, 26], making applications in the second molar region particularly challenging. Furthermore, d-CAIS necessitates separation between hand and eye coordination [27, 28]; moreover, the substantial size of the handpiece [13] used in d-CAIS might complicate its clinical application in this area.

In this study, we utilized an autonomous robotic system with the following operational characteristics: The robotic arm can autonomously perform osteotomy and implant placement, but the surgeon is required to maneuver the robotic arm into and out of the patient’s oral cavity. During the robotic arm insertion process, to prevent alteration of the pre-calibrated approach axis, the system is designed to lock the handpiece orientation. This design feature makes it challenging to directly position the handpiece in restricted-access surgical sites with limited mouth opening.

Therefore, we describe clinical technique that enables the application of r-CAIS in second molars. When transitioning to manual mode, the operational state closely resembles freehand implantation, allowing surgeons to manipulate the robotic arm to ensure a flexible path of the drills into the osteotomy hole. Once properly positioned, the system was switched to automatic mode to enable the robotic arm to perform the subsequent surgical procedures.

However, since the implant osteotomy and placement are still performed automatically by the robotic arm, the impact on surgical accuracy is minimal. This is also reflected in the surgical precision data we obtained. Although the automatic-manual mode switching function was employed, the surgical accuracy of robot-assisted second molar procedures was less than 0.5 mm at both the coronal and apical deviations, and the angular deviation was less than 1°. In a retrospective study focused on robot-assisted implant placement, the mean coronal, apical and angular deviations are 0.44 ± 0.17 mm, 0.46 ± 0.17 mm and 0.85 ± 0.47°, which is consistent with our findings [29].

The proximity of the maxillary second molar root apex to the maxillary sinus floor [30], as well as the close distance between the mandibular second molar root apex and the inferior alveolar nerve canal [31], significantly elevates surgical risks during implant surgery in this region. The high precision of the r-CAIS enhances safety during operations in this area. In this study, four maxillary second molar implants (cases 1–4) were placed close to the floor of the maxillary sinus, whereas 12 mandibular second molar implants (cases 5–16) were placed close to the inferior alveolar nerve canal at a safe distance (>2 mm). This is probably associated with the good depth control of the robotic arm. As shown in Table 2, the mean vertical apical deviation (−0.04 mm) was significantly lower than the mean lateral apical deviation (0.36 mm). This finding aligns with various r-CAIS studies [20, 32, 33]. This improved depth control may be closely related to real-time intraoperative monitoring of depth, as illustrated in Fig. 6c, which allows surgeons to obtain a clear indication of depth by observing the computer screen. This feature has the potential to reduce the conventional 2 mm safety threshold in the posterior mandible or avoid sinus lift in some borderline situations in the posterior maxilla [34–36], thereby reducing surgical risks.

It has been demonstrated that step drills effectively prevent surgeons from deviating from the previous axis during subsequent preparation [37]. In vitro studies have indicated that, unlike straight drills, the implementation of step drills can mitigate the placement deviations associated with s-CAIS [37]. The step drills achieve this function because the diameter of the drill tip is the same as the previous drill body. In this study, we used step drills combined with manual-automatic mode switching to help complete implant placement in second molars with r-CAIS. This technological advancement has the potential to help with the application of r-CAIS in more intricate clinical situations in the future, particularly for patients with mild to moderate limited mouth opening.

Nonetheless, several limitations exist. First, the preparation time for robotic surgery is relatively long. The process necessitates marker installation, CBCT scanning, and surgical plan design, which increase patient discomfort. Future studies should focus on simplifying the clinical procedures to minimize the preparation time. Second, this study only assessed placement accuracy and did not evaluate patient satisfaction or long-term clinical outcomes; thus, extended follow-up observations are warranted. Third, no measurements of individual patients’ mouth opening were documented, and the possible correlation between mouth opening extent and implantation precision remains unexamined. Finally, the sample size of this study was small; further prospective studies with large sample sizes are essential to validate the reliability of this technology.

## Conclusions

r-CAIS, when utilized with a manual-automatic mode switching function and step drills, can accurately place implants in the second molar region with limited operating space. Further prospective studies with larger sample sizes are needed to validate the effects.

## Data Availability

The data that support the findings of this study are available from the corresponding author upon reasonable request.

## Abbreviations

CBCT: Cone-beam computed tomography
s-CAIS: Static computer-assisted implant surgery
d-CAIS: Dynamic computer-assisted implant surgery
r-CAIS: Robotic computer-assisted implant surgery
DICOM: Digital Imaging and Communications in Medicine
